# The influence of rewards on (sub-)optimal interleaving

**DOI:** 10.1371/journal.pone.0214027

**Published:** 2019-03-18

**Authors:** Christian P. Janssen, Emma Everaert, Heleen M. A. Hendriksen, Ghislaine L. Mensing, Laura J. Tigchelaar, Hendrik Nunner

**Affiliations:** Experimental Psychology and Helmholtz Institute, Utrecht University, Utrecht, The Netherlands; Middlesex University, UNITED KINGDOM

## Abstract

We investigate how the rewards of individual tasks dictate a priori how easy it is to interleave two discrete tasks efficiently, and whether people then interleave efficiently. Previous research found that people vary in their ability to interleave efficiently. Less attention has been given to whether it was realistic to expect efficient interleaving, given the reward rate of each of the involved tasks. Using a simulation model, we demonstrate how the rewards of individual tasks lead to different dual-task interleaving scenarios. We identify three unique dual-task scenarios. In easy scenarios, many strategies for time division between tasks can achieve optimal performance. This gives great opportunity to optimize performance, but also leads to variation in the applied strategies due to a lack of pressure to settle on a small set of optimal strategies. In difficult scenarios, the optimal strategy is hard to identify, therefore giving little opportunity to optimize. Finally, constrained scenarios have a well-defined prediction of the optimal strategy. It gives a narrow prediction, which limits the options to achieve optimal scores, yet given the structure people are able to optimize their strategies. These scenarios are therefore best to test people’s general capability of optimizing interleaving. We report three empirical studies that test these hypotheses. In each study, participants interleave between two identical discrete tasks, that differ only in the underlying reward functions and the combined result (easy, difficult, or constrained scenario). Empirical results match the theoretical pattern as predicted by simulation models. Implications for theory and practice are discussed.

## Introduction

Multitasking is prevalent in today's society and is therefore studied in many disciplines (see special issue in [1]). Various studies have investigated how good people are at multitasking, with a focus on performance decrements compared to single-task settings (e.g., [2–5]). This helped to identify settings where, when given a choice, multitasking should be minimized, such as in the car. However, in practice, to multitask or not is not always a choice, but a given: air traffic controllers [6], doctors [7], and office workers all multitask. The choice in such situations is then how to divide a finite amount of time between multiple tasks (cf. [8]).

An important subset of all multitasking scenarios is task interleaving, in which a person stops working on one task (temporarily) in favor of another task, potentially to later return to the initial task [9] (see also section “How interleaving relates to other multitasking settings”). Task interleaving happens frequently. For example, office workers tend to interleave tasks every two to three minutes, with many self-initiated task interleaving [10–12], and some programmers even interleave every 20 to 120 seconds [13]. An open question then is how efficient such interleaving practices are? Before being able to assess this in applied contexts such as office work, first requires a general understanding of the factors that affect interleaving efficiency.

For example, is it efficient to start marking exams while your computer restarts? This depends on the type of exam questions. Open questions take long to mark initially, as you grow into the marking process. With time, you get faster at marking, almost in an exponential way. However, by then your computer has restarted and you might want to continue computer work. In contrast, closed questions (e.g., multiple choice) can be marked fast, almost at a linear rate. As there is no long "investment" phase, it is efficient to start marking these questions.

This example reflects a general property of diverse tasks. Tasks have specific reward functions and reward rates (e.g., exponential versus linear) that influence our decisions to interleave. However, what is lesser known is how different combinations of such rewards dictate the opportunity of people to interleave efficiently (i.e., to optimize the amount of rewards that are obtained across both tasks within a finite time frame). Once situations have been identified in which people have an opportunity to interleave efficiently, people’s general ability to do so can be tested.

In this paper, we investigate how the rewards of individual tasks dictate whether people have an easy, difficult, or constrained opportunity (defined in more detail later) to divide their time between two tasks efficiently. We then also test people’s ability to achieve optimal performance in these scenarios in three studies. Given the novel nature of studying rewards in interleaving scenarios, and the relevance of interleaving for everyday settings (e.g., [9,10–13]), we focus on a controlled version of interleaving two discrete tasks.

More specifically, experiment 1 tests opportunity and ability to optimize behavior in a situation without time pressure. Experiment 2 introduces a time limit to create time pressure. Due to the time pressure, picking the right strategy for interleaving becomes essential, because each task switch comes at the cost of precious time and missed opportunity. Experiment 3 has an even further reduced time limit, to create further time pressure. In addition, the number of trials is extended to see whether additional experience reduces any observed sub-optimality (due to ceiling effects). Before introducing these studies in more detail, we will first motivate the design based on a review of related work on the use of rewards in task interleaving settings.

### Studies of rewards

Many multitasking and task interleaving studies use rewards (e.g., [14–18]). However, only few looked at how reward functions affect our ability to multitask efficiently. These studies found that participants tried to maximize rewards, and thereby optimized performance [19–24]. However, in these studies, the optimal scores were not achieved in all conditions by all participants. For example, some participants transferred strategies from previous conditions to new conditions without extensive exploration of better alternatives, thereby satisficing, and not optimizing, performance [24,25]. Further, the research by Farmer et al. [24] suggested that the ability to optimize also varied between different reward functions. Specifically, for reward functions where many interleaving strategies could result in losses, participants seemed to act risk averse (cf. [26].)

A limitation of this previous research was that only a restricted set of reward functions has been studied. A systematic comparison of theoretically identified reward alternatives is missing, as is an understanding of how variations in these rewards limit the opportunity to multitask efficiently. Our aim is to provide such a principled study of the effect that reward functions have on dual-task interleaving behavior.

**Fig 1:**
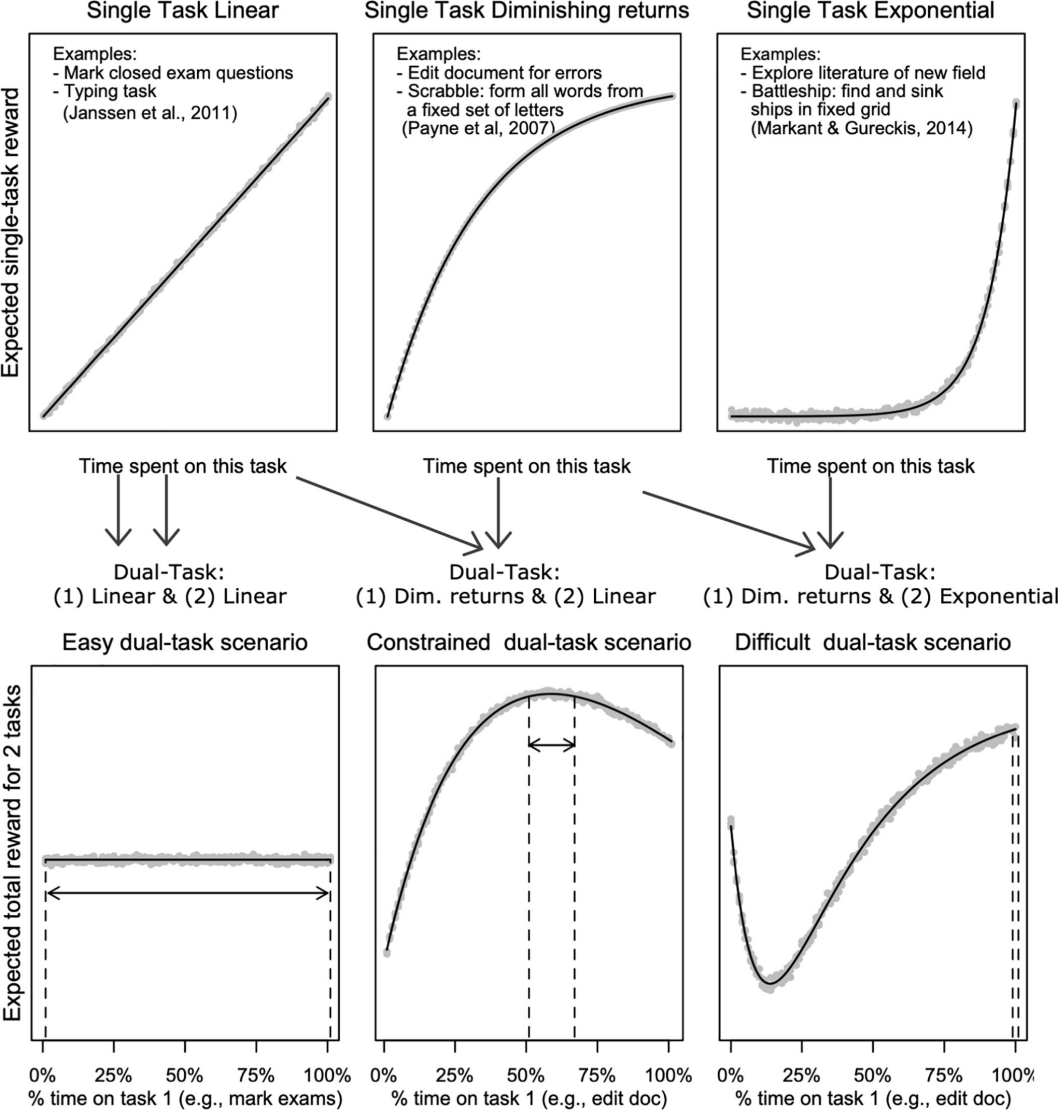
Illustration of the general problem. Top: Single-tasks differ in their underlying reward functions. Bottom: Different dual-task scenarios emerge from different task combinations. Black lines show mean expected rewards of random trials (grey dots) given the strategy (time on task). Strategies within the dashed lines achieve optimal rewards (within 1% of maximum). The combination of single-tasks into dual-tasks impacts the a priori chance of finding an optimum interleaving strategy.

Von Winterfeldt and Edwards [27] identified that most everyday single-task settings can be approximated using one of eight possible reward functions. These functions describe how performance (e.g., speed, accuracy) and associated (monetary) rewards are expected to increase with time investment. Three reward functions are of special interest, as they are applied in our current studies: linear, exponential, and diminishing return rewards. These are also illustrated in the top row of Fig 1.

For linearly increasing rewards, the amount of rewards that a task provides remains relatively constant over time and increases at a constant pace. An example is marking closed exam questions, such as multiple-choice exams. Per time unit, roughly the same number of questions can be corrected, and each question will be equally valuable. Many rote tasks have similar characteristics. Linear rewards have been used in dual-task experiments, for example in settings where participants had to type in constant strings of digits (e.g., [21,24,25]).

Exponentially increasing rewards are an example of a bigger class of reward functions in which early investments are required to achieve an eventual bigger payoff. An example is exploration of literature from a new domain for a literature review. This is initially hard, as there is little frame of reference for understanding the details of the literature. However, over time one’s knowledge grows, new literature can be understood more easily, and cross-connections between papers can be made. To the best of our knowledge, few studies have systematically used exponential rewards in dual-task studies. However, they have been implicitly applied in other tasks. For example, in the game of battleship [28], a player needs to sink an opponents’ ships by systematically exploring positions on a board. Initially, it can take long to find part of a boat (initial investment). However, once part of a boat has been identified, it can be sunk quickly (eventual payoff).

For diminishing return rewards, reward rate also changes over time. However, in such settings the initial steps give the most rewards, and with each next step the amount of reward per step is reduced. An everyday example is document editing. Initially, it might be easy to edit a written document (e.g., many errors to correct, sentences to improve), which improves the quality of the manuscript and thereby its potential rewards. However, on subsequent editing rounds the amount of improvements that can be made reduces severely, because most, if not all, errors have been corrected already. Tasks with diminishing returns have been used in two dual-task settings that implicitly had this reward structure. Duggan and colleagues [19] used an office task in which documents needed to be corrected and rewards were given for each completed document. Initial documents only required quick fixes, but the amount of corrections increased for each subsequent document, thereby diminishing the number of rewards over time. Similarly, Payne and colleagues studied scrabble like puzzle tasks [22,23]. Here rewards are also diminishing, as initially the generation of a set of words given a fixed set of letters (e.g., LNAOIET) comes quickly (e.g., TEA, TOE, TEN, LANE), but over time it becomes harder to think of additional new words.

More generally, diminishing returns functions are frequently found in literature on animal foraging [29] and human foraging (e.g., searching information on the web [30,31]). Although foraging theory has been applied to dual-task scenarios [19,22,23], this literature has only used diminishing return rewards. Instead, our work looks at situations in which reward rates can also remain constant over time (linear) or increase over time (exponential). How does this influence the decision to remain on a task, or to switch?

As will be demonstrated next, these three single-task rewards (linear, exponential, diminishing) can provide interesting dual-task scenarios. Von Winterfeldt and Edwards [27] identified five other options, which we do not explore further in this study. Three of the other options are situations in which points are lost over time following either a linear, exponential, or diminishing return function. These are not used, as our focus is on situations where working on a task leads to gains. The two other functions are reward functions that are U-shaped (in which the reward rate first drops over time, and then increases again) or inverse-U shaped (in which the reward rate first increases over time, and later drops over time). Again, given our focus on situations with gains, these functions were less suitable for our study.

### Combining rewards in dual-task settings

In a discretionary dual-task setting, there are two tasks between which time is divided. The three single-task reward functions that we identified can create six dual-task combinations (i.e., linear + linear, linear + diminishing, linear + exponential, diminishing + diminishing, diminishing + exponential, exponential + exponential). We focus on three of these scenarios, that differ in an interesting way in their predictions for dual-task behavior. The three scenarios are also illustrated in the bottom row of Fig 1. For each scenario, we define the expected gain as a function of strategies for interleaving. Strategies can be conceptualized in various ways. Within the context of the current paper, we define it as the proportion of time that is spent on each of the tasks.

1. In Easy Interleaving Scenarios, many strategies result in optimal rewards (Fig 1: near 100%). For example, when grading two exam piles with identical closed questions, roughly the same number of questions gets marked using different interleaving strategies (e.g., marking a complete pile before switching, or occasionally interleaving between the two piles). Given that many strategies result in optimal performance (i.e., there is lots of opportunity to optimize interleaving), we hypothesize that people quickly achieve optimal performance. Moreover, as there is no need to prefer specific strategies over other strategies, we hypothesize that there is variation within individuals and between individuals in the strategies that they apply. Note that in practice switching between tasks takes time and too frequent switching can affect performance in the easy scenario–this is addressed in experiment 2 through introduction of a time limit.
2. In Difficult Interleaving Scenarios, the optimum strategy is difficult to find. An example is interleaving editing a manuscript for typing errors (diminishing returns) with exploring literature from a new field (exponential rewards). Initial investment in the diminishing returns task can provide some quick wins, however, this is in conflict with the necessity of the exponential task to make early investments before getting potentially higher returns on investment later. This makes it hard to sacrifice one task for the other. More generally, this class of settings might not have a clearly identified global maximum, but might have multiple local maxima (e.g., as in Fig 1). We hypothesize that in difficult interleaving scenarios, people have difficulty to achieve optimal performance, as the optimal strategy is not easily identifiable. Therefore, the optimal strategy is not identified, or only after extensive experience. As a consequence, there is again variation within and between participants in the strategies that are applied, as they might not know what strategy is best and continue searching for a better alternative. That said, in practical situations, they might satisfice [32] and settle on local optima.
3. In Constrained Interleaving Scenarios there is a narrow, well-defined prediction of the optimum strategies (e.g., in Fig 1: only 17% of strategies). More specifically, we call a scenario constrained when it meets these three criteria: (a) There is one global maximum, (b) There are no local maxima other than the global maximum, (c) when the strategies for interleaving are ordered in a systematic way (for example: based on proportion of time spent on a task) there is a coherent, small set of strategies that achieves the highest reward. An example is interleaving document editing (diminishing return rewards) with marking a closed question exam (linear rewards). Initially, great strides can be made on the document editing, but when marginal improvement on the writing reduces, one should let it rest and mark exams. By comparison to the difficult condition, the constrained condition has some structure to it which makes it easier to find the optimal strategy. We therefore hypothesize that participants find the optimal strategy faster in this condition compared to the difficult condition, but slower compared to the easy condition. As the constrained condition has a small set of optimal strategies, we also expect that these strategies, once found, are applied more consistently within and between participants compared to the easy condition. More generally, given the well-defined prediction of optimality in constrained scenarios, it provides a perfect setting for testing people's general ability to multitask efficiently. In contrast to the easy scenario, finding the optimal strategy is not trivial and requires deliberate strategies.
  What constitutes small is subjective. Within our study this can be explicitly defined due to the use of models. The intuition is that if a model were to apply strategies at random, it should only end up in the optimal set in few instances (e.g., less than 5% of the cases). This test of where a random model would end up ensures that it is not trivial to end up at the optimal strategy, and differentiates this category from the easy interleaving scenarios.

With these three scenarios, we can revisit our two research questions. First, the opportunity that a dual-task scenario offers to optimize interleaving performance differs between easy, difficult, and constrained interleaving scenarios. Therefore, it allows us to compare human performance under different conditions.

Second, to demonstrate human general ability to optimize dual-task performance, some settings are better test cases than others. As explained above, the constrained scenario provides the ideal scenario to test optimality: there is a concrete set of strategies identified, that is small enough to be falsified experimentally (cf. [33]). Observing optimality in the easy condition does not provide solid evidence for general ability, given the wide set of opportunities that this setting offers. By contrast, the difficult condition provides a too strict test for general optimality, as there are very specific factors that limit human’s general ability.

### Other factors influencing optimality

Of course, other factors than rewards can also affect dual-task interleaving strategies, but compared to studies of the influence of rewards, these other factors have been reported in much more detail in the literature, and have been incorporated in other detailed cognitive models of tasks (e.g., see [34] for cognitive models of various multitasking settings, and [35] for various models of multitasking while driving). For example, task interleaving is affected by task difficulty [36], task structure [37], motivation [38], cues [39,40], reward uncertainty [41], and switch costs [42]. These aspects are controlled for in the current experiment, to allow focus on the effect of rewards.

In addition, the exact parameters of the reward functions also matter. For example, for linear tasks it matters whether the task gives very few points per step (e.g., 0.5 point per step) by comparison to the other task (e.g., 50 points per step). This will change the predictions of where the exact optimum time division lies and to what degree the emerging dual-task scenario is more like an easy, constrained, or difficult case. In our study, we have set the parameters in such a way that a fair comparison between the scenarios can be made. Each single-task can give at most 100 points, whereas each of the chosen dual-task case can be clearly categorized as one of the identified scenarios (easy, difficult, constrained). Such a clear division of scenarios is needed to allow future investigation of more complicated combinations. To allow such investigations, we have provided the code of experiment 1 and experiment 3 and our model and analyses code as supplementary material, together with the data from the experiment.

### How interleaving relates to other multitasking settings

We have cast our study as discretionary task interleaving, which involves switching between discrete tasks, where the person only works on one task at a time. Interleaving is a subclass of the wider class dual-tasking and multitasking, which can also include working on two or more tasks in parallel.

Interleaving also has similarities with task switching. However, task switching has a more narrow and specific common interpretation in the form of the classical task switching paradigm [42]. In this paradigm, a person works on isolated and independent gives a judgement response to stimuli (e.g., higher/lower than 5 or odd/even) on consecutive, independent trials. In the voluntary task switching paradigm (e.g., [43–45]), participants can decide what type of task they want to respond to (odd/even or high/low), whereas in the classical (non-voluntary) paradigm the type of judgement is dictated by the experiment(er) [42]. Our setting differs from task switching in that we do not have fully independent trials. Instead, participants respond to a stimulus (“whack a mole”, see method), but the resulting score depends on how often they have responded to that task before.

Finally, the stopping rule from the foraging literature can be used to emphasize that one decision that a participant makes is when to stop working on a task [22,29–31]. Indeed, as will be demonstrated in the studies, in our set-up one fruitful strategy is to work on one task until limited further progress can be made (e.g., points diminish) and to then fully stop working on this task and continue to another task. Similar to earlier dual-task studies that are grounded in foraging theory [19,22,23], we label our situation as a class of task interleaving, as the person does have the option to return to an original task. Similar to that earlier work, the optimal decision might be not to return to the original task, however, the person has the option to do so if they desire.

## Experiment: Whack-a-mole task

**Fig 2:**
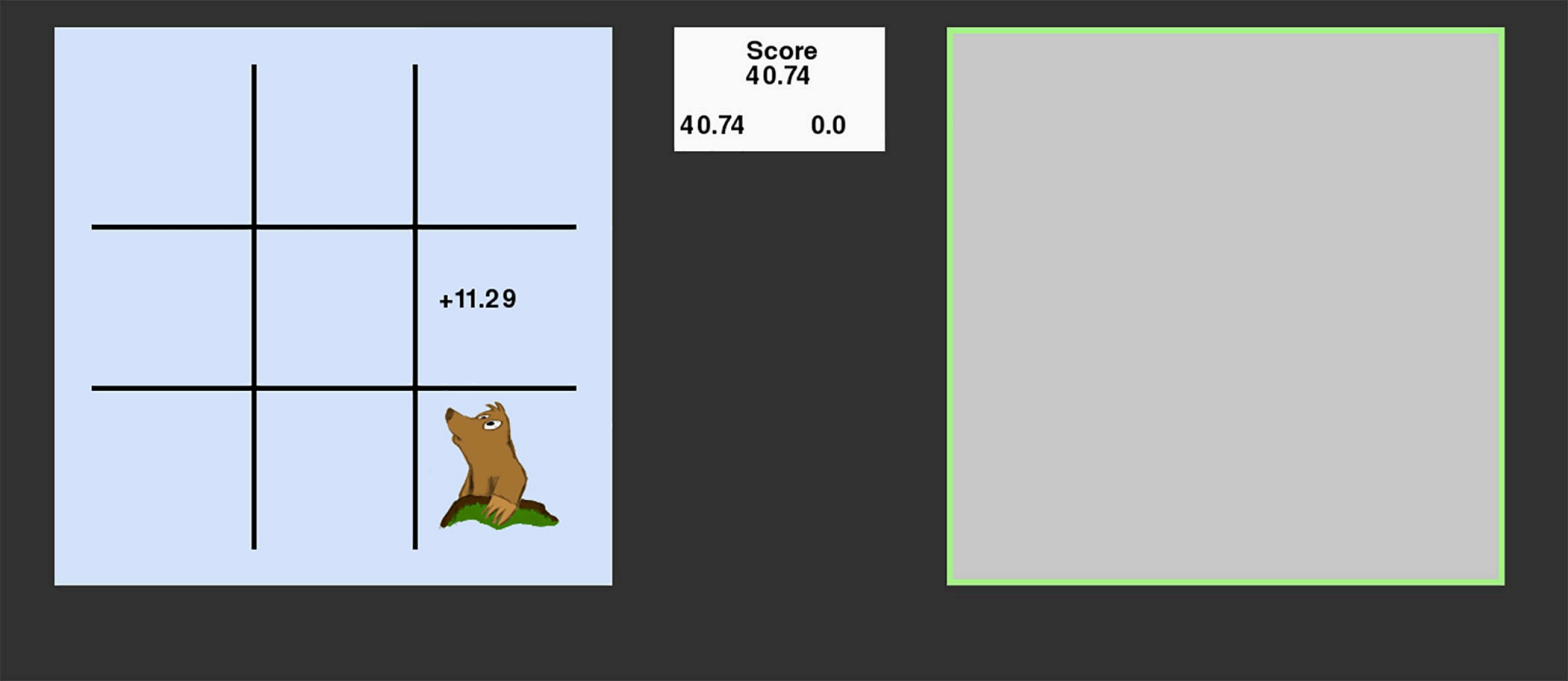
A screenshot from the Whack-A-Mole task in the dual trial.

The focus of our experiment is to understand how the pattern for interleaving two tasks is affected by the underlying reward functions. Therefore, participants interleave between two identical tasks that only differ in the rewards that they provide. The main task is to “Whack moles” that appear on the screen in a 3 x 3 grid. Once a mole appears, it only disappears when a corresponding key on the numeric keyboard is hit (e.g., key 2 for the bottom-middle mole, see Fig 2). Each mole provides points. In total, participants have to hit 50 moles. However, participants can decide themselves how many moles they hit on each of the two tasks (left task, right task).

Each task has its own reward function. For example, when the task underlies a linear function, it provides 2 points per mole; with a diminishing function the points per mole decrease with each mole that is ‘whacked’ (e.g., 16.00, 13.44, 11.29, … 0.003 points). A trial ends after 50 moles are wacked (all studies). For studies 2 and 3 a trial also ends after a time limit has passed.

The study controls for known factors that influence task interleaving strategies. First, effects of task difficulty are avoided by using two identical tasks (while keeping the manipulation of dual-task scenario and associated theoretical difficulty). Second, the task consists of small steps that are consistent, such that any influence of patterns in the task structure and ‘natural breakpoints’ for interleaving [37] is avoided. Third, uncertainty about rewards can hinder optimization [41]. Therefore, we provide participants with explicit feedback on their achieved score after each mole (e.g., “+2”) and keep counters of the total score per screen and combined. Moreover, within each block of trials the function that underlies the scoring of a task is kept constant, this is explained to participants and is also reflected in a consistent color coding of the background of the task (see materials for details). Fourth, motivation affects performance [38], and therefore we created a task that is similar to a classic arcade game. Moreover, we awarded a gift card to the best performing participants in each study. Finally, switching between tasks takes time, and potentially also mental switch costs [42]. To minimize the switch costs, we kept the task simple: it did not rely on memory. Moreover, in experiment 1 we did not use a time limit, such that switching between tasks did not take precious time that could be spent on ‘whacking moles’. In experiment 2 and 3 we do introduce a time limit so temporal switch costs become important. The code of experiment 1 and experiment 3 is provided as supplementary material.

## Experiment 1: Interleaving without time pressure

### Method experiment 1

#### Participants

Fifty participants took part on a voluntary basis. Two participants were excluded because of software crashes. The final sample included 48 participants (21 males; 27 females), between 20 and 27 years of age (*M* = 21.69 years, *SD* = 1.65 years). For one participant (participant 2) some data of the last trial is missing due to a logging error. Participants received compensation in the form of study credit points. In addition, the highest scoring participants entered a lottery to win one of two gift cards, worth €10,-. The experiment was approved by the ethics committee of the Faculty of Social and Behavioral Sciences of Utrecht University (approval number FETC15-020). All participants gave written informed consent prior to the experiment.

#### Materials

The experiment was developed in Python 2.7.9. It was displayed on a HP 1740 (1280x1024 pixels) monitor with a HP keyboard with enabled numeric keypad to register responses. Participants viewed the screen from a distance of approximately 70 cm and only used the numeric keypad, arrow keys, and the space bar of the keyboard.

The general task was to ‘whack’ moles that appear on the screen in a 3 x 3 grid. Once a mole appears, it only disappears when a corresponding key on the numeric keyboard is hit (e.g., key 3 for the bottom-right mole, see Fig 2). Each mole provides points. In total, participants have to hit 50 moles. In the dual-task trials, participants need to decide how to divide their time between two such tasks, so as to achieve the optimal combined score.

For each task, one of three reward functions was used. In all conditions, a 100 points total could be earned when all 50 moles in that task were wacked. In the linear condition, each mole that was wacked gave the participant 2 points. In the diminishing condition, the first mole gave 16.00 points, and from then the amount of points systematically diminished (13.44, 11.29, 9.48, … 0.003 points). Conversely, in the exponential condition, the first mole gave 0.031 points, and from then the amount of points exponentially increased (0.036, 0.040, 0.045, … 11.336). The background color of the task differed per reward function. The colors were beige (RGB: 179, 89, 0) for linear tasks, light green (136, 255, 136) for diminishing return tasks, and light blue (109, 103, 237) for exponential tasks.

Each time when a participant wacked a mole, the screen would display a score at the location of the wacked mole (e.g., +11.29 in Fig 2). Moreover, this score would be added to the score field at two locations: to the total score (at the top of the white area in Fig 2), and at the lower score field that kept track of the total score per screen (e.g., for the left task in Fig 2, the bottom left score is updated).

If participants typed an incorrect key, the mole would not be whacked. Such typing errors did not have direct consequences for the participant’s score, other than that they spent time on something that would not help them progress in the task. The supplementary material of this manuscript contains an analysis of typing errors.

During each dual-task trial, the experiment showed two task screens next to each other. Both were covered by a grey square, but showed a border in the color of the reward condition. At the trial start, participants had to press the arrow key of the screen they wanted to open first (left or right arrow). This would reveal that task, and allowed participants to whack the moles in this task. If participants wanted to switch to the other task, they had to press the space bar. This would cover the current window with a grey square (only showing the border) and reveal the other task (see Fig 2). The task would continue until 50 moles in total were wacked.

In the experiment we combined different reward functions to achieve our three desired dual-task scenarios. In the easy condition, two linear functions were combined. In the constrained condition, the linear function and the diminishing returns function were combined. In the difficult condition, the exponential function and diminishing returns function were combined. An analytical model was used to determine what scores could be achieved by participants, based on how many moles were hit on each task. The shape of these curves differs between conditions. In the easy condition, all interleaving strategies (i.e., alternatives for proportion of time spent on each task) achieve 100 points (50 x 2). In the constrained condition there was a gradual but steep increase in points in the direction of the optimal strategy (whacking 38 moles on the linear task, and 12 on the diminishing task), which resulted in a maximum score of 163.6733 points. Twelve strategies (23.4%; typing between 8 and 19 moles on the diminishing task) achieve a score that is within 10% of maximum score, relative to the possible range of scores. In the difficult condition, the score increase is less steep, and the optimal strategy (7 moles on diminishing; 43 on exponential) achieves 113.5323 points. This task has 6 strategies (11.8%; typing between 5 and 10 moles on the diminishing task) that achieve a score within 10% of the maximum score, relative to the possible range of scores.

Finally, a questionnaire with demographic questions and questions to document participants subjective experience of success (documenting how successful they think they were in each condition).

#### Design

We used a one-way within-subjects design with three levels, representing the dual-task scenario: easy (linear + linear task), constrained (diminishing + linear), and difficult (diminishing + exponential).

To avoid effects of the location of a task, we counterbalanced the following factors. First, we counterbalanced the order in which the dual-task conditions were offered (6 unique orderings). Second, we counterbalanced whether the diminishing task (when presented) was provided on the left or the right side. Third, we counterbalanced whether during the single-task trials participants first encountered the left task, or the right task. This gave 24 (6 x 2 x 2) unique counterbalanced conditions, each of which was performed by two participants.

#### Procedure

Upon arrival, participants were briefed about the study and given time to sign the informed consent form. Participants were first made familiar with the task by practicing two single-task trials and one short dual-task trial (e.g., to learn how to whack a mole, how to switch tasks).

This was followed by three experimental blocks. In each block, participants first performed each task (left and right) once in a single-task version in which they experienced how the reward for each mole changed over time (e.g., linear, exponential, or diminishing), without receiving explicit information on the shape of the function from the experimenter. In each block, there were then fifteen dual-task trials. On each trial, 50 moles needed to be hit, but the participant was free to determine how many moles they hit on each of the tasks (i.e., what proportion left, what proportion right). The last five trials were used to calculate a mean score across conditions to determine who achieved the highest score and therefore was eligible for the gift card. Participants were told that they could use the first 10 dual-task trials to explore various strategies and to exploit this during the last 5 trials.

The total score was given at the end of each trial, with an average score given every fifth trial (of the score over last five trials). Participants had to press space to start the next trial.

After the experiment, the demographic questionnaire data were collected. Finally, participants received a debriefing and the option to take a written debriefing home with them. The total procedure took approximately 75 minutes.

#### Measures

The software logged each keypress of the user as well as changes in the user interface. In addition, we calculated using an analytical model what the possible range of scores is, and what the maximum achievable score is in each condition. Based on this logging and the model, we analyze the strategies that participants apply and how these compare with model predictions of the optimum strategy. The data from all experiments and an analysis script are included as supplementary materials. For one participant (participant 2) a logging error lead to not having all data of the last trial, hence this trial is not used in the analysis.

### Results experiment 1

We counted how frequently participants switched between windows on each trial for the last five trials of each condition. Table 1 reports the cumulative count across participants for all three experiments. For experiment 1, the majority of the participants does not switch between windows on most trials of the easy task (where both tasks always reward equally). For the constrained and difficult condition, the majority of participants switches once. However, in all conditions there are also observations where participants switch more frequently.

**Table 1:** Number of switches between windows per experiment.

	Number of switches from one task/window to the next						
	0	1	2	3	4	5	>5
**Experiment 1 (48 participants; 240 trials per condition)**							
Easy (linear + linear)	174	34	13	2	2	4	11
Constrained (linear + diminishing)	7	208	8	9	3	1	4
Difficult (diminishing + exponential)	14	189	23	5	1	1	6
**Experiment 2 (23 participants; 115 trials per condition)**							
Easy	97	14	4	-	-	-	-
Constrained	10	97	4	4	-	-	-
Difficult	23	74	9	5	4	-	-
**Experiment 3 (20 participants; 100 trials per condition)**							
Constrained	-	94	2	3	1	-	-
Difficult	1	91	5	3	-	-	-

Count of number of trials where there was a switch from one window to the next per experiment and per condition. For this table, we only included data from the last 5 trials of that condition. For ease of reading “-”indicates that no observations of that category were made.

As there was no time pressure in this task, switching between windows did not have an explicit penalty, apart from that the participant took longer to finish the experiment. The more interesting question is how they divided their time between the two tasks: How many moles did they whack on each task? To investigate this, we analyzed how time was divided between the windows of the two tasks.

**Fig 3:**
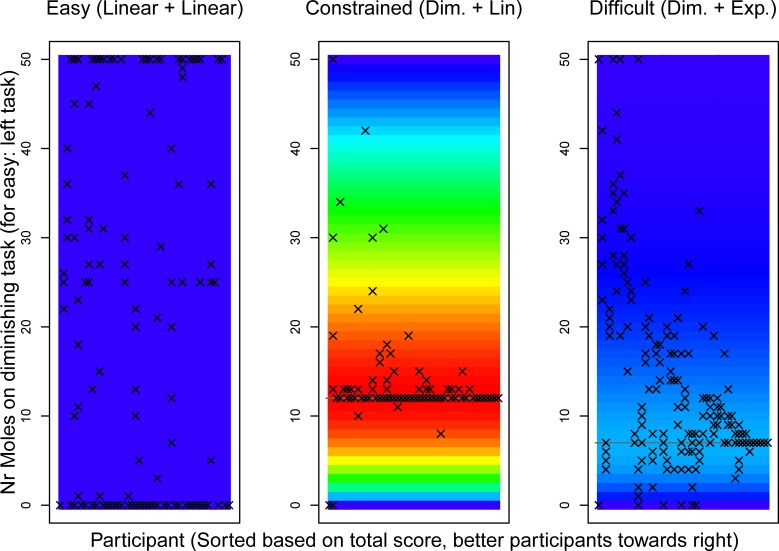
Heatmaps of the predicted score for interleaving tasks at a specific division with human data. For the easy task, the y-axis shows the number of moles whacked on the left task, for the other two scenarios the y-axis shows the number of moles whacked on the tasks with diminishing return rewards. Crosses show the strategies that participants applied during the last five trials of each scenario. Participants (horizontal axis) are sorted based on their total score for the trials that contributed towards their score, with better participants towards the right. In the easy and difficult scenario there is variation in the strategies that are applied between and within subjects. In the constrained scenario there is little variation, as participants found the optimal strategy and applied this.

Fig 3 plots the chosen strategy (number of moles on one of the tasks; vertical) for each participant (horizontal) for the three conditions (three plots). A heatmap indicates what scores are possible for the various strategies. The warmest red color is given for the highest score (163. 6733 points on the constrained task) and the coldest dark blue color is given for the lowest score (100 points). The colors are distributed evenly between these extremes. For the constrained and exponential task, the vertical axis shows the number of moles hit on the diminishing returns task. Crosses mark the performance of each participant during the last five trials. The participants are sorted based on their total summed score from the last five trials of each condition, with higher scoring participants to the right.

In the easy condition, participants varied in the strategies that they apply between trials, as there was no incentive to optimize. The large majority of participants (41 out of 48, or 85.4%) had at least one trial in which they focused on only one of the tasks (i.e., whacked 0 moles on one task, 50 on the other). Twenty-five participants (52.1%) did this on all five trials, yet did vary per trial whether they focused on the left or right task.

In the difficult condition, few participants achieved the optimum score. Twenty participants (41.7%) applied the strategy that achieved the optimal score at least on one trial, but only 5 participants (10.4%) did so consistently on all trials. Instead, the majority of participants applied different strategies on different trials. Our interpretation is that these participants had not yet found the optimal strategy, as this was a difficult scenario.

In the constrained condition, scores were better by comparison. Specifically, 40 participants (83.3%) applied the optimal strategy on at least one trial, and 23 participants (47.9%) applied the optimum strategy within the first 5 trials of the constrained condition. By comparison, in the difficult condition only 9 participants (18.8%) achieved the highest score within 5 trials, and another 7 (14.6%) achieved it within the first 10 trials.

### Experiment 2: Time limit

The results of experiment 1 demonstrate that the reward function of tasks strongly impacts participants’ a priori opportunity to optimize their performance. In the easy and constrained condition, application of the optimum strategy is more plausible compared to the difficult condition. In the easy condition, this is trivial by definition and the lack of a pressure to apply a specific strategy leads to variation in strategies between and within individuals. In the constrained condition there is also optimal performance, but this time with more consistent strategies being applied, given that there is clear differentiation in scores between strategies and there is a clear optimum strategy (compared to the difficult condition).

One open question is whether this pattern remains when a time limit is introduced. Specifically, due to the time limit, it becomes costly to elaborate long about what strategy to apply, and it becomes costly to switch between tasks (i.e., a switch cost). Indirectly, the time limit then introduces time pressure. Does the same pattern of behavior remain?

Looking into time pressure is of practical importance, as many situations require people to perform tasks within a finite amount of time. For example, office workers might need to respond to e-mails within a specific number of hours or days. From a theoretical perspective, studying the effect of a time limit and imposed time pressure is also important. For example, dual-process models of cognition ([46,47], see also [48]) posit that behavior under time pressure can significantly differ from behavior without time pressure. The typical interpretation is that behavior under time pressure is driven by relatively more automatic or intuitive responses, whereas without time pressure there is more room for reflective and deliberative responses. The effects of time pressure have therefore been studied in many domains and setting, such as cooperation [49,50], altruism [51], honesty [52,53], and social value orientation [54]. Our contribution is to study how time pressure affects interleaving in a dual-task setting: what dual-task scenarios do still allow achieving optimal interleaving performance?

### Method experiment 2

The method of the experiment was the same as experiment 1, with the following changes.

#### Participants

Twenty-three participants took part on a voluntary basis (7 male; 16 female). Ages ranged between 19 and 27 years of age (*M* = 21.78, *SD* = 2.28). The two participants with the highest scores were rewarded a 10 euro gift card. The experiment was approved by the ethics committee of the Faculty of Social and Behavioral Sciences of Utrecht University (approval number FETC15-020). All participants gave written informed consent prior to the experiment.

#### Materials

The task was run on different hardware: a MacBook Pro 10.7.5 (1280 x 1024) with a Dell keyboard. The software was the same, with the exception that each dual-task trial ended either after 50 moles were hit, or after 30 seconds, whichever came first.

#### Design

We used a one-way within-subjects design with three levels, representing the dual-task scenarios: easy (linear + linear task), constrained (diminishing + linear), and difficult (diminishing + exponential). We were one participant short of fully counterbalancing dual-task conditions (6), where the diminishing task was presented (left or right) and which task was practiced first (left or right). Although our intention was to collect data on a fully counterbalanced design, time requirements and low subject availability due to summer did not allow us to do so.

#### Procedure

The procedure was the same as experiment 1 with the exception that there was a time limit of 30 seconds and participants were informed of the time limit.

#### Measures

All measures were the same. However, due to the time limit, the number of moles that participants could whack was affected by individual typing speed and the number of times that participants switched between the two tasks. We therefore developed a process model to predict for each individual the impact of interleaving strategy on expected score. This model is described next.

## Process model of interleaving strategies

We developed a model that can predict expected score for various strategies of how time is divided between two tasks for each individual. The R code of the model is provided as supplementary material to this paper.

The model first systematically goes through all possible number of moles that can be wacked on one of the tasks (ranging between 0 and 50). Given the strategy, it would calculate the time needed to (1) whack all moles on the first task, (2) switch between tasks, and (3) whack moles on the other task.

### Initial typing and whacking of moles

For any given strategy, the model assumes that the number of moles that is defined by the strategy is whacked if trial time allows. The associated time cost (perceiving the mole, considering what digit to type, and typing it) is calibrated to the times that were observed for each individual in the experiment. For each participant, we calculated their median interkeypress interval and the standard deviation of their interkeypress interval for intervals between two consecutive correct keypresses/whacks on the dual-task trials. For the experiment, these median values ranged between 387 and 576 ms in experiment 2 (*M* = 498 ms, *SD* = 47 ms), and between 360 and 509 ms in experiment 3 (*M* = 443 ms, *SD* = 36 ms). In the model, on each trial, when a new mole was whacked, we sampled a value from a normal distribution with the mean set to the median interkeypress interval of that participant and the standard deviation as observed. If the sampling procedure sampled a value below 50 ms, a new sample was sampled, to avoid inclusion of negative values.

### Switching windows

For simulations that switched between the two windows, we estimated the average switch cost for individuals. This was estimated by calculating for each individual the average time interval between finishing typing a digit on one task and starting to type a digit on the other task. The average across individuals was taken and then corrected by the average median time needed to type a digit. This resulted in a switch cost of 739 ms for experiment 2, and of 491 ms in experiment 3.

### One model run

For each individual model run, we assumed that a model either did not switch (for the strategy that typed as many digits as possible on one task), or switched once or twice between the windows. For models that switched, the model first calculated the total time needed for the switch(es). Then the model calculated how many digits could be typed in total, by sampling typing times from the modeled typing distribution of a specific individual while there was remaining trial time. Once a trial was finished, the model first attributed the number of digits typed to one of the task as dictated by a strategy, and the remainder to the other task. For example, if the strategy was to whack 49 moles on task A and 1 on task B, a model that managed to whack 50 in total during the trial, would whack 49 on A and 1 on B. A model that only managed 49 would whack 49–0. A model that only achieved 48 would whack 48–0.

### Calculating payoff

With the resulting prediction of trial times and number of moles whacked on each task, the payoff function of the experiment could be used to calculate what the achieved score would be under all possible payoff functions.

### Simulation procedure

For each individual, each unique strategy, and each number of switches (0 for strategies that typed all in one window, or 1 or 2 switches for other strategies) we ran 100 simulations to get a stable estimate of the expected average score of the strategy.

### Results experiment 2

Table 1 reports how frequently participants switched between windows during the last five trials. The pattern is similar to that of experiment 1: most participants on most trials did not switch between windows on the easy task, and switched once for the difficult and constrained task. Due to the added time pressure, we have fewer or no observations of trials in which participants switch frequently, when compared to experiment 1 (no time pressure).

**Fig 4:**
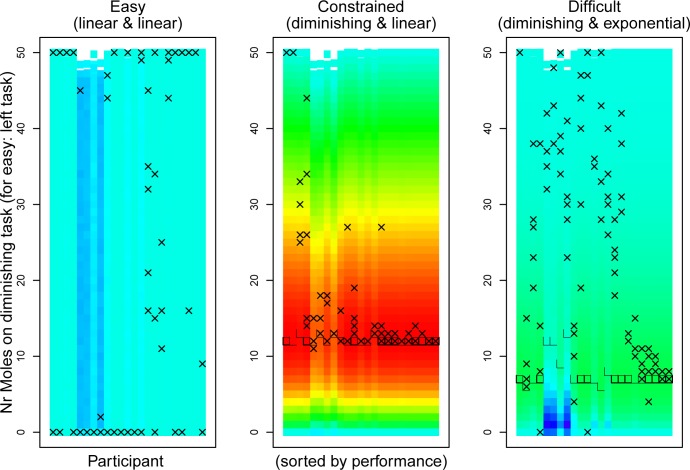
Heatmaps of predicted scores with achieved scores in Experiment 2. The heatmap follows the same format as Fig 4. Square boxes indicate where the most efficient strategy was for the constrained and difficult condition. The pattern is largely the same as before: in the constrained condition there is more consistency in the applied strategy, and most participants apply the efficient strategy.

Fig 4 plots a heatmap of predicted model scores for all strategies compared to the strategies that participants applied, with participant data, and participants sorted based on their total achieved score during the critical trials (last five of each condition). The location of the optimal strategy occasionally varies between participants. This is due to variations in typing speed. For example, in the difficult condition the highest rewards on the exponential task are only received after typing many digits, whereas rewards on the diminishing task are given early on. Therefore, participants that type relatively slow should not “risk” going for the late rewards on the exponential task, as (due to their slow typing) the time limit might be hit before they achieve these critical late high points.

In the easy condition, participants varied in the strategies that they applied between trials, as there was no incentive to optimize. For slower typing participants (the 5^th^, 6^th^, and 8^th^ in Fig 4 for example), there was some slight variation in score between strategies as these participants did not manage to type all 50 trials, and between simulations it varied how many they could type exactly.

In the constrained condition, 16 of the 23 participants (69.6%) applied their global optimum strategy at least once, of which 5 (21.7%) applied it consistently on all trials. Two participants (8.7%) consistently only whacked moles on one of the tasks (first two participants in Fig 4). One of these did so also in the other two conditions, and debriefing revealed that this participant had not understood the instructions (as she asked when the dual-tasking part started).

In the difficult condition, only 4 participants (17.4%) applied their global maximum strategy at least once, but not consistently in all trials. The location of the optimum also varied more between slower and faster typers, with slower typers having to focus more on the diminishing returns task. Compared to the other two conditions, in particular the constrained condition, there was more variation in the applied strategies between and within participants.

One way in which participants seemed to have tackled the challenge of a time limit is by typing faster in dual-task compared to single-task. A paired t-test found that participant had speeded up typing in dual-task (*M*_*median*_ = 498 ms, *SD*_*median*_ = 47 ms, range = [387, 576]), compared to the single-task trials (*M*_*median*_ = 526 ms, *SD*_*median*_ = 62 ms, range = [404, 636]), *t*(22) = -3.74, *p* = .001.

### Experiment 3: Time limit and more trials

The aim of experiment 2 was to see whether the behavioral pattern observed in experiment 1 replicated when a time limit, and associated switch cost and potentially time pressure, got introduced. The pattern largely replicated and supports our conclusion that the payoff function determines a priori how likely it is to optimize performance in a discretionary dual-task situation. The interleaving strategies that participants apply varies more between and within participants either if there is no need to settle on a strategy to achieve optimal performance (easy scenario), or if the optimum is hard to find (difficult scenario). In a constrained scenario, participants can find the optimum, although compared to experiment 1 a slightly larger percentage of participants does not find the optimum.

In experiment 3 we test whether the fact that we observed few uses of the optimal strategy in the difficult condition (and for some, in the constrained condition) might be alleviated by more experience: if the number of trials is increased from 15 to 25 per payoff condition. In addition, we also reduced the total trial time to 25 seconds. The aim behind this change was to have more participants that would not be able to whack all 50 moles in one trial, and therefore to have more variation in the location of the optimum strategy between participants. In experiment 2, participants typed faster in dual-task trials compared to single-task trials, which might be an adaptation to the enforced time pressure.

### Method experiment 3

The method of the experiment was the same as experiment 2, with the following changes.

#### Participants

Twenty-one participants took part on a voluntary basis (16 male; 5 female). Ages ranged between 20 and 36 years of age (*M* = 24.47, *SD* = 3.31). The post-study questionnaires revealed that one participant completely misunderstood what the experiment was about (they thought the experiment was on eye-hand coordination), and did not attempt any task interleaving. This participant was therefore excluded from data analysis. Twenty participants remained for the analysis. The experiment was approved by the ethics committee of the Faculty of Social and Behavioral Sciences of Utrecht University (approval number FETC15-020). All participants gave written informed consent prior to the experiment.

#### Materials

The task was run on two different hardware set-ups. Sixteen participants did the task on a desktop PC (AMD FX-6300 3.50GHz six-core processor; 8 GB RAM; Windows 7 Enterprise—Service Pack 1). Five participants ran the task on a Macbook Pro. Both set-ups used identical keyboards with a numeric keyboard and nearly identical 22” Dell monitors. For the efficiency of data collection, sometimes two participants were ran in the same room, but on different sides of the room and outside of each other’s sight.

There were only two experimental blocks: a constrained block and a difficult block. For each block the number of dual-task trials was 25. Before trial 21, participants were informed that these trials counted towards the calculation whether they achieved the optimal score. Each trial had a time limit at 25 seconds.

#### Design

We used a one-way within-subjects design with two levels, representing the dual-task scenario: constrained (diminishing + linear), and difficult (diminishing + exponential). We attempted to counterbalance dual-task conditions (2 options), where the diminishing task was presented (left or right), and which task was practiced first (left or right). This gave 8 unique situations. We were four participants short from testing each of these situations three times (fully counterbalanced), however the order of conditions and whether the right or left window was opened was counter-balanced.

#### Procedure

The procedure was similar to experiment 2, except that we only had 2 dual-task blocks instead of 3, and that each block had more trials (25 instead of 20). The total duration was approximately 60 to 75 minutes.

### Results experiment 3

As in experiment 1 and 2, the large majority of participants switched only once between the two windows per trial (see Table 1). Also similar to experiment 2, the participants speeded up their typing in the dual-task trials compared to the single-task trials. A paired t-test found that participant had speeded up typing in dual-task (M_median_ = = 443 ms, *SD*_*median*_ = 36 ms, range = [360, 509]) compared to the single-task trials (*M*_*median*_ = 508 ms, *SD*_*median*_ = 68 ms, range = [403, 695]), *t*(19) = -4.79, *p* < .001. The mean value in the dual-task trials is around 50 ms shorter than the value for dual-task trials in experiment 2, suggesting that the participants adapted to the further time pressure by typing faster.

**Fig 5:**
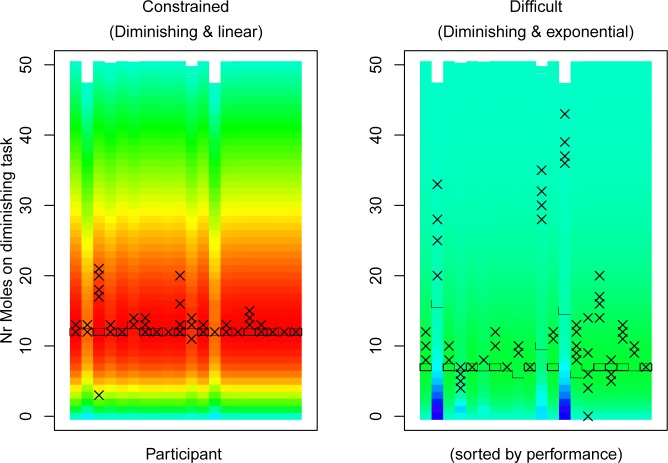
Heatmaps of predicted scores with achieved scores in Experiment 3. The heatmap follows the same format as Fig 4. Despite the even shorter time limit, the majority of participants still performs efficient in the constrained condition.

The heatmaps in Fig 5 show that in the constrained condition 18 participants (90%) applied their optimal strategy at least once, of which 8 (40%) applied it consistently on all five trials. All participants but one applied strategies that were in the warmest zone of the heatmap on all of the last trials (the exception is the third participant in Fig 5).

In the difficult condition, 5 participants (25%) applied their personal optimal strategy at least once, and 4 (20%) did so consistently. The majority of participants spent relatively more time on the diminishing returns task than on the exponential task (i.e., higher values on the vertical axis of Fig 5). This can be considered a risk-averse strategy: with the exponential task the high rewards only came later in the experiment, but due to the time pressure there was some uncertainty whether those could be achieved.

**Fig 6:**
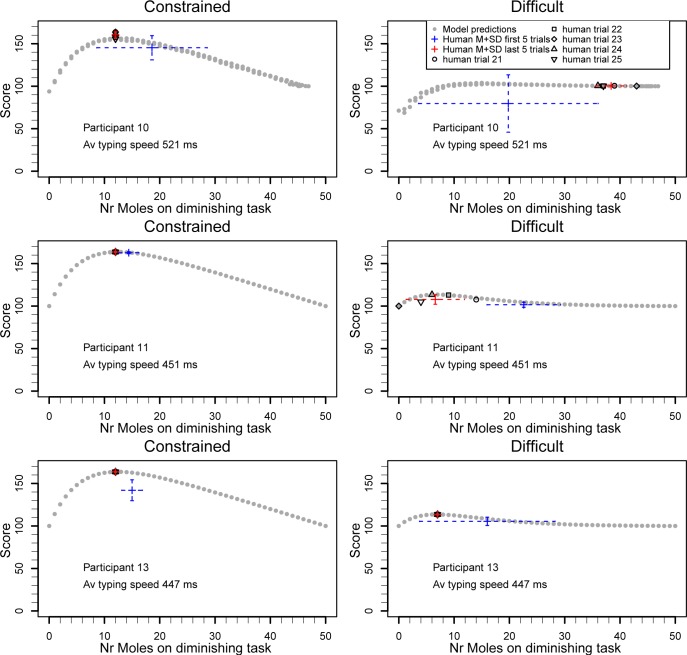
Illustration of the individual pay-off curves of 3 participants (rows) in two payoff conditions. The shape of the curve changes based on typing speed, especially in the difficult condition. Participants change their strategies with experience, and based on the nature of their individual pay-off curve.

As an illustration, Fig 6 shows the individual pay-off curves for three participants in both conditions. In general, the model predictions and human achieved performance aligned well. Notice how for the relatively slower typing participant (top two panels of Fig 6), the shape of the payoff curve was slightly different, with a less defined bump. It shows the benefit of being risk averse: the achieved score by spending most time on the diminishing returns task (i.e., on the right of the horizontal axis in Fig 6) is quite close to the actual optimal score.

## General discussion

This paper investigated two questions: (1) how reward functions a priori dictate the ability to optimally interleave two discrete independent tasks, and (2) whether people are able to interleave optimally. Considering how rewards impact interleaving ability is of theoretical interest given the diversity of claims that exist regarding multitasking and task interleaving efficiency and optimality [2–5,20,21,24,25]. A difference in reward structure between tasks might provide one explanation why previous studies differ in their observations of (sub-) optimality. Moreover, task interleaving occurs frequently in practice, for example office workers switch every two to three minutes between discrete tasks [10], and some programmers even interleave every 20 to 120 seconds [13]. Our study can inform under what reward conditions frequent interleaving might be (in-)efficient.

We found that at least three dual-task interleaving scenarios can occur, based on combinations of tasks with different reward functions. In easy scenarios (for example, a combination of two task with comparable linear rewards), there is a large range of strategies that achieves optimal performance. We found consistently that participants varied in the strategies that they applied to interleave tasks in easy scenarios, as there is no reason to settle on a specific strategy. Because easy scenarios allow many ways to achieve optimal performance by definition, such scenarios have no strong predictive power about people’s general ability to optimally interleave two tasks in other contexts. Therefore, they are of limited use for studies that want to make theoretical claims about general efficiency and optimality. For practice (e.g., in office settings), easy scenarios are only desirable in cases where efficiency is desired, but convergence and consistency in behavior is not required.

In difficult scenarios (for example, combining exponential rewards with diminishing return rewards), aspects of the reward functions make identification of the optimum a priori hard. In our scenario, the difficulty came from the conflicting nature of the two reward functions, where the diminishing return requires time investment (to gain the early high rewards), which conflicts with the needed time investment in the exponential rewards (to later gain high rewards). In other contexts, difficulty can also arise due to other characteristics such as local, non-global optima that compete with the global optimum. For difficult scenarios, we found consistently that there is also variation in strategies between and within participants, but due to a different cause: the optimum strategy has not been identified. In experiment 3, participants had more trials to explore the strategy space, and a slightly larger percentage of participants eventually found the optimum strategies. However, the set of participants that found the optimum strategy was smaller in the difficult scenario compared to the constrained scenario. Difficult scenarios are therefore of limited value for theoretical claims about optimality, as a priori it is unlikely that people achieve this. For practice they are also unsuitable, as people are unlikely to perform well.

Finally, in constrained scenarios there is a clear optimum, which is not at a trivial position (i.e., not spending all time or 50–50 on each task), and in which there are no elements from a difficult scenario such as conflicting rewards or local maxima. In the constrained scenario from our experiment, the large majority of participants applied the optimum strategy on at least one trial, and often did so consistently towards the end of the experiment. This suggests that participants can learn to divide their time efficiently in these constrained scenarios. Therefore, such scenarios provide ideal test beds for theoretical studies of general task interleaving efficiency: they can test ability without the prediction for optimal behavior being trivial (as in easy), or unrealistically hard (as in difficult scenario). Moreover, such scenarios are interesting for practical situations (e.g., office work) in which little variation in performance is desired.

Interestingly, studies by Payne, Duggan, and colleagues that investigated the efficiency of discrete task interleaving in various scenarios such as puzzle solving [22,23] and manuscript editing [19] used scenarios that can be classified as “constrained” (in these studies both tasks had diminishing reward functions). Consistent with our results, these studies also observed that people were relatively efficient. This supports our claims that (1) constrained scenarios are best to test general optimality of task interleaving, and (2) that people can in principle be optimal when put in situations that are not too easy or too difficult. Our theoretical framework of rewards provides an explanation of why these previous experiments worked.

### Implications

The main theoretical implication of our work is that studies of task interleaving, especially when focusing on efficiency and optimality, should give careful, explicit consideration to the reward functions that are inherent to the task. We suggest that scenarios that are like our constrained scenario are best to test general claims of optimality. Moreover, our findings suggest that people can indeed interleave efficiently in such situations, in line with earlier work [19,22,23]. Difficult scenarios might be useful in identifying a subset of participants that is relatively efficient compared to the average population, but should not be used to make claims about people’s general ability to optimize.

Regarding time pressure, previous work focusing on dual-process theories of cognition posit that behavior can be different under time pressure ([46,47], see also [48]). The typical interpretation is that under time pressure participants’ ability to achieve the “rational” solution to a problem is diminished. Our results refine this view. We demonstrate that for dual-task scenarios, the ability to interleave in an optimal manner depends, at least in part, on the reward functions that underlie the tasks, and the type of dual-task scenario that emerges from it. In easy and constrained scenarios, the majority of participants was still able to apply the optimal interleaving performance when placed under time pressure due to a time limit. Therefore, it is not the case that all interleaving performance is diminished under dual-task conditions.

The main practical implication of our research is that people can flourish most under constrained conditions and are likely to perform poorly under difficult task interleaving scenarios. Settings where task interleaving is prone to happen, such as office environments [10], and some engineering settings [13], should therefore strive to facilitate such environments when assigning tasks to employees. In difficult scenarios, additional training or experience might be needed to learn how to efficiently balance time, given the small effect that more experience might have had in experiment 3. In addition, in settings where the opportunity for training and extra experience is limited, other resources might be needed. For example, recent work has looked at the effect of gamification to encourage people to complete difficult tasks [55]. In our context, this might be interpreted as applying an additional reward function to make a difficult scenario look more like a constrained scenario (e.g., making the experience of rewards on an exponential task more like the rewards on a linear task, by rewarding early actions disproportionately large).

Easy, difficult, and constrained scenarios are easy to differentiate in our task paradigm, yet in practice there might be situations where a categorization is less clear cut, because it meets criteria of more than one scenario (e.g., has characteristics of a constrained and difficult scenario). This is not a problem if the study does not want to make claims about interleaving efficiency or optimality. However, in situations where the study wants to make claims about optimality, and in which the paradigm itself is not essential (e.g., it is not a study about “driving” in which inclusion of a driving task is essential), we suggest to change the task design to have a clearer differentiation in scenarios.

### Limitations and future work

In each experiment, there were some participants that did not achieve optimal behavior in the constrained task. For some, this was due to a misinterpretation of instructions. However, in general this opens up further investigation of what makes some participants better than others. Given the small number, we have not yet found consistent predictors. Our models open up opportunities to investigate this further, as they provide an objective, explicit way for identifying sub-optimal participants.

We have only investigated three dual-task scenarios, with a limited set of reward functions (and their associated specific parameters), as this set was enough to identify three general interesting classes: easy, difficult, and constrained scenarios. There is a possibility that other classes of dual-task scenarios exist that we have not yet identified. A specific limitation in this regard is that our reward functions all had a cap of 100 points (when all 50 moles were whacked on that task). There can be practical situations in which there is no such cap, leading to more differentiation between tasks. For example, situations where exponential rewards give disproportionately large rewards, beyond a fixed maximum, as one progresses towards the end of a task. We therefore do not claim that we studied all possible dual-task scenarios, but we do claim that we studied an interesting subset. In addition, we have not looked at settings where more than two tasks need to be interleaved, as it was not even known whether efficient behavior can be observed in the simpler case of dual-task interleaving scenarios.

Another interesting class of payoff functions to look at is functions that “reset” when the associated task is not attended to. In such cases, the potential patterns of interleaving become more dynamic. In contrast, in the current set-up, although some participants interleave frequently between tasks, frequent interleaving is not needed to achieve the highest score. Moreover, when participants are placed under time pressure they reduce how frequently they interleave (see also Table 1). Having functions “reset” when not attended can change this dynamic. For example, if a diminishing return reward function resets to its initial high values each time when it is revisited, this might encourage more frequent interleaving. If an exponential reward resets to its initial low values, it might discourage frequent interleaving.

A different type of setting is when the value of a task diminishes when the task is not being attended too. This occurs in driver distraction scenarios, or other situations in which there is task progression even in the absence of attention to the task. Our previous research suggests that under some conditions participants can be efficient in these scenarios [37] and that the nature of the reward function also influences performance [24,25]. Situations that include interdependencies between tasks require more advanced models to predict human behavior.

We provided explicit feedback to participants about the rewards that a single mole gave, and on the accumulated score per task and across the two tasks. Moreover, there was no uncertainty about whether rewards were awarded after each action. Reducing the feedback, or introducing uncertainty about the payoff might result in longer learning times [41]. Therefore, a difficult scenario might become even harder to solve, and it might take longer for participants to find the optimum interleaving pattern in a constrained scenario. Nonetheless, the relative differences between such scenarios should remain: by comparison, a constrained scenario is the preferred environment to test claims about interleaving efficiency.

The reward values in our task were communicated explicitly and tasks were similar across the different reward functions. In practice, different tasks have different reward functions inherently. It is an open question how efficient people are in such situations, as other factors that have been studied in detail before also start affecting interleaving pattern, including task difficulty [36], task structure and the occurrence of natural breakpoints in tasks [37], motivation [38], and switch costs [42]. That said, data suggests that at least tasks that confirm to a constrained scenario show an ability to interleave efficiently between two discrete tasks [19,22,23].

## Supporting information

S1 File Experiment code files experiment 1. The zip-file contains the code to run the experiment in the version of experiment 1 (no time limit, Dutch instructions).
(ZIP)

S2 File Experiment code files experiment 3. The zip-file contains the code to run the experiment in the version of experiment 3 (time limit, English instructions).
(ZIP)

S3 File Model and analysis script and data. The zip-file contains three Rdata files with all the data from the three experiments, 2 Rdata files with simulated model results for simulations of experiment 2 and experiment 3 and the R code to analyze the data and to run model simulations for each experiment.
(ZIP)

S4 File Supplementary analysis of typing errors. (DOCX)
