# Supplementary material for: The influence of rewards on (sub-)optimal interleaving
Source: PLoS One. 2019 Mar 18;14(3):e0214027. doi: 10.1371/journal.pone.0214027 (PMC6422303; doi:10.1371/journal.pone.0214027)
Supplement: S1 File — The zip-file contains the code to run the experiment in the version of experiment 1 (no time limit, Dutch instructions). (ZIP) [file pone.0214027.s001.zip › howToRunCodeExperiment1.rtf]

How to Python code experiment 1:Note that this version of the experiment has DUTCH instructions. The code for experiment 3 gives instructions in English.To run the code, you need to have installed Python, and the following packages (some of which come in the combined package “Anaconda”):pygamemathscipy(see also the top of the file behind “import”)These packages work for example with Python 2.5.4 on Mac.Before being able to run the program, make sure that the directory in which the code is stored also contains the files:- “EmmamolScaled.png” a picture of the mole to be wacked- “participantConditions.csv” this is a file from which the code reads which participant to expose to which condition in which orderThe code works best in combination with a numeric keyboard (to “whack the moles”)To run:- Open the python code (experiment1_code.py) with IDLE. - Select “Run module” in the main menu. If all went well, you see no error messages (if you see error messages, it might be that packages or files are missing)- type in the command window:    main()- enter a participant number and type enter (the number should be one of the numbers listed in the “participantConditions.csv” file)The experiment will start. The experiment saves a .csv file with the participant data in its working directory (the directory in which the code is also)If you want to check out the code in detail, search for “def main()”, which will lead you to the main function. From there you can see which other functions are being called.
