# Supplementary figures and images for: The influence of rewards on (sub-)optimal interleaving
Source: PLoS One. 2019 Mar 18;14(3):e0214027. doi: 10.1371/journal.pone.0214027 (PMC6422303; doi:10.1371/journal.pone.0214027)

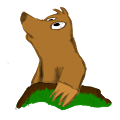

Supplement: S1 File — The zip-file contains the code to run the experiment in the version of experiment 1 (no time limit, Dutch instructions). (ZIP) [file pone.0214027.s001.zip › EmmamolScaled.png]
