# Supplementary material for: The influence of rewards on (sub-)optimal interleaving
Source: PLoS One. 2019 Mar 18;14(3):e0214027. doi: 10.1371/journal.pone.0214027 (PMC6422303; doi:10.1371/journal.pone.0214027)
Supplement: S4 File — (DOCX) [file pone.0214027.s004.docx]

## S4 Supplementary analysis of typing errors

Typing errors had no direct influence on the point score of the participants (i.e., no points were subtracted). However, typing an erroneous digit does take time. For experiments 2 and 3, this can indirectly affect score as it might prevent participants from being able to whack all moles.

We analyzed what proportion of the digits was incorrect (i.e., fraction incorrect keypresses out of the total number of keypresses) and express that as a percentage below. The table below summarizes this data for each experiment across all trials:

**Table Sup1.**

| Experiment | Mean | Minimum | 1^st^ quantile | Median | 3^rd^ quantile | Maximum |
| --- | --- | --- | --- | --- | --- | --- |
| 1 | 4.8 | 0.7 | 2.1 | 3.6 | 6.5 | 16.3 |
| 2 | 8.6 | 2.9 | 4.7 | 6.2 | 12.6 | 19.1 |
| 3 | 15.5 | 1.6 | 5.5 | 9.7 | 13.6 | 67.8 |

Percentage of typing errors across all trials

In general, the large majority of participants has a low percentage of incorrect keypresses. The introduction of a time limit (experiment 2) and setting a stricter time limit (experiment 3) seems to shift the distribution more upwards, with the 1^st^, 2^nd^ and 3^rd^ quantile increasing (i.e., they result in a relatively higher percentage of typing errors).

One participant in experiment 3 (participant 4) had an excessively high percentage of incorrect keypresses (67.8 – that is, they typed more incorrect digits than correct digits).

We repeated the analysis for the subset of the data that only contained the last five trials of each condition, as this subset of the data is also used in other analyses. The range of values is roughly in the same ballpark as analyzed before.

**Table Sup2.**

| Experiment | Mean | Minimum | 1^st^ quantile | Median | 3^rd^ quantile | Maximum |
| --- | --- | --- | --- | --- | --- | --- |
| 1 | 4.6 | 0.5 | 2.0 | 3.0 | 6.3 | 18.7 |
| 2 | 8.6 | 2.6 | 4.6 | 6.7 | 12.3 | 19.8 |
| 3 | 15.6 | 1.8 | 6.5 | 7.6 | 13.9 | 68.1 |

Percentage of typing errors across the critical trials (last five trials of each condition)

We also studied how this participant faired in terms of score on the experiment. In Figure 5, data of all participants is shown individually, with the data sorted based on their score on the last five trials of the two conditions. Participant 4 is the 3rd participant that is shown in this Figure. In other words, it is one of the lower scoring participants. Indeed, on the majority of trials the participant did not apply the optimal strategies.
